# Intracellular β_2_-adrenergic receptor signaling specificity in mouse skeletal muscle in response to single-dose β_2_-agonist clenbuterol treatment and acute exercise

**DOI:** 10.1007/s12576-013-0253-z

**Published:** 2013-03-13

**Authors:** Shogo Sato, Ken Shirato, Ryosuke Mitsuhashi, Daisuke Inoue, Takako Kizaki, Hideki Ohno, Kaoru Tachiyashiki, Kazuhiko Imaizumi

**Affiliations:** 1 Laboratory of Physiological Sciences, Faculty of Human Sciences, Waseda University, 2-579-15 Mikajima, Tokorozawa, Saitama 359-1192 Japan; 2 Department of Molecular Predictive Medicine and Sport Science, Kyorin University, School of Medicine, 6-20-2 Shinkawa, Mitaka, Tokyo 181-8611 Japan; 3 Department of Natural and Living Sciences, Graduate School of Education, Joetsu University of Education, 1 Yamayashiki, Joetsu, Niigata 943-8512 Japan; 4 Global COE Doctoral Program, Graduate School of Sport Sciences, Waseda University, 2-579-15 Mikajima, Tokorozawa, Saitama 359-1192 Japan

**Keywords:** β_2_-Agonist clenbuterol, Exercise, Skeletal muscle, β_2_-Adrenergic receptor signaling, p38 MAPK, Akt

## Abstract

The aim of this study was to clarify the intracellular β_2_-adrenergic receptor signaling specificity in mouse slow-twitch soleus and fast-twitch tibialis anterior (TA) muscles, resulting from single-dose β_2_-agonist clenbuterol treatment and acute exercise. At 1, 4, and 24 h after single-dose treatment with clenbuterol or after acute running exercise, the soleus and TA muscles were isolated and subjected to analysis. The phosphorylation of p38 mitogen-activated protein kinase (MAPK) increased after single-dose clenbuterol treatment and acute exercise in the soleus muscle but not in the TA muscle. Although there was no change in the phosphorylation of Akt after acute exercise in either muscle, phosphorylation of Akt in the soleus muscle increased after single-dose clenbuterol treatment, whereas that in the TA muscle remained unchanged. These results suggest that p38 MAPK and Akt pathways play a functional role in the adaptation to clenbuterol treatment and exercise, particularly in slow-twitch muscles.

## Introduction

β_2_-Adrenergic receptor (β_2_-AR) signaling in skeletal muscles plays a role in the regulation of skeletal muscle hypertrophy and metabolism. Numerous studies have shown that the selective β_2_-agonist clenbuterol, a doping drug, induces skeletal muscle hypertrophy, transformation of muscle fibers, and alterations in muscle metabolic enzymes [1, 2]. On the other hand, exercise has generally accepted to elicit multiple signals to heighten muscle protein synthesis, fiber-type switching, and mitochondrial biogenesis [3]. These adaptations to exercise are mediated in part by β_2_-AR activation [4]. However, the effects of β_2_-agonist clenbuterol and exercise on the activity of β_2_-AR signaling pathways in skeletal muscles are unclear. Studying β_2_-AR signaling in skeletal muscles is able to identify potential therapeutic applications for muscle wasting conditions such as sarcopenia, cancer cachexia, denervation, and neuromuscular diseases, aiming to attenuate the muscle wasting and associated muscle weakness, and to enhance muscle growth and repair after injury [1, 2].

It is known that β_2_-AR can couple to both Gα_s_ and Gα_i_ signaling pathways. The Gα_s_-adenylyl cyclase (AC)-cyclic adenosine monophosphate (cAMP) pathway is the best characterized of the β_2_-AR signaling pathways. Phosphorylated protein kinase A (PKA) induced by cAMP regulates the activity of proteins, including cAMP response element binding protein (CREB) [1, 2]. CREB is a nuclear transcription factor that is ubiquitously expressed and has been implicated in cell proliferation, differentiation, adaptation, and survival [5]. In addition to the well-documented inhibition of AC activity, β_2_-AR coupling to Gα_i_ appears to activate Gα_s_-independent pathways, such as mitogen-activated protein kinase (MAPK) and phosphoinositol 3-kinase (PI3K)-Akt signaling pathways [1, 2, 6–8]. The MAPK family, including extracellular signal-regulated kinase 1/2 (ERK1/2), c-Jun N-terminal kinase (JNK), and p38 MAPK, is a ubiquitous group of signaling proteins involved in the control of cell growth, differentiation, and adaptation [9]. In particular, p38 MAPK activity in skeletal muscles is necessary for myogenic cell differentiation [10] and plays a role in glucose metabolism and energy expenditure [11, 12]. The PI3K-Akt pathway has also been implicated in protein synthesis, gene transcription, cell proliferation, and cell survival [13].

On the basis of metabolic and contractile properties and myosin heavy chain isoforms, skeletal muscle fibers can be broadly classified as type I, oxidative, slow-twitch fibers and type II, glycolytic, fast-twitch fibers. We have previously reported that the changed expression of β_2_-AR mRNA resulting from clenbuterol treatment for 10 days in rats [14, 15] and removing muscle loading by arthrodesis of rats for 10 days [16] is dependent upon muscle fiber type. On the basis of these findings, we hypothesized that the response of intracellular β_2_-AR signaling to clenbuterol treatment and exercise may differ between muscle fiber types. Therefore, in the present study, we investigated the acute effects of clenbuterol treatment and exercise on the phosphorylation of CREB, p38 MAPK and Akt in mouse skeletal muscles to clarify the intracellular signaling specificity in the typical slow-twitch soleus and fast-twitch tibialis anterior (TA) muscles.

## Materials and methods

### Experimental animals

Male 10-week-old C57BL/6J mice were obtained from CLEA Japan (Tokyo, Japan). These mice were housed in a temperature (23–25 °C) and humidity (50–60 %)-controlled room with a 12-h light–dark cycle. Regular rodent laboratory chow and water were available ad libitum. The experimental procedures and animal care were approved by the Committee on Animal Care, Ethics and Use, Waseda University (2011-A003), and conducted according to the Guiding Principles for the Care and Use of Animals in the Field of Physiological Sciences, the Physiological Society of Japan.

### Experimental protocols

#### Acute experiment

For the β_2_-agonist experiment, clenbuterol hydrochloride (Sigma, St. Louis, MO, USA) dissolved in 0.9 % NaCl solution was injected intraperitoneally (i.p.) into mice (1.0 mg/kg body weight). At 1, 4, and 24 h after treatment, the soleus and TA muscles were isolated. For the exercise experiment, because moderate exercise has been previously defined as brief (usually 15–60 min) bouts of treadmill running at 50–75 % maximum O_2_ consumption or 15–22 m/min [17], mice were subjected to treadmill running at 18 m/min for 1 h. At 1 (just after exercise), 4, and 24 h after exercise, the soleus and TA muscles were isolated. In all experiments, skeletal muscles were rapidly removed from mice anesthetized with isoflurane (Mylan, Tokyo, Japan), frozen in liquid nitrogen, and stored at −80 °C.

#### Chronic experiment

Mice were subjected to 6-week voluntary exercise, β_2_-agonist clenbuterol treatment, or both. The animals were randomly divided into 4 groups: sedentary control (S) group, voluntary exercise-trained (EX) group, clenbuterol-treated (CL) group, and EX + CL group. EX and EX + CL mice were singly housed in stainless steel cages (height 80 mm, width 90 mm, depth 220 mm) and given 24-h access to a running wheel for 6 weeks. Wheel revolutions were counted daily and multiplied by the wheel circumference (628 mm) to obtain daily running distances. S and CL mice were singly housed in similar cages without a running wheel for 6 weeks. Further, CL and EX + CL mice were injected i.p. with clenbuterol (1.0 mg/kg body weight/day; Sigma) dissolved in 0.9 % NaCl solution. S and EX mice were also injected i.p. with the same volume of 0.9 % NaCl solution. All mice were sacrificed at 24 h after the last voluntary exercise or injection. Skeletal muscles were rapidly removed, frozen in liquid nitrogen, and stored at −80 °C.

### Extraction of cytosol and membrane protein in skeletal muscles for measuring β_2_-AR expression

The measurement of β_2_-AR expression in cytosol and membrane fractions of skeletal muscles has been described previously [16, 18]. Briefly, frozen muscles were homogenized in ice-cold homogenization buffer (0.3 M KCl, 0.1 M KH_2_PO_4_, 50 mM K_2_HPO_4_, and 10 mM EDTA; pH 6.53, 1:20, w/v) containing a protease inhibitor cocktail (Thermo Fisher Scientific, Rockford, IL, USA). The homogenate was centrifuged at 100,000*g* for 1 h at 4 °C. The resultant supernatant was collected as the cytosol fraction. The pellet was homogenized in an ice-cold solubilization buffer (homogenization buffer containing 1 % Triton X-100; 1:20, w/v). After incubation for 2 h, the homogenate was centrifuged at 100,000*g* for 1 h at 4 °C. The resultant supernatant was collected as the membrane fraction. Each protein sample was subjected to sodium dodecyl sulfate–polyacrylamide gel electrophoresis (SDS-PAGE) and transferred onto a polyvinylidene fluoride (PVDF; Millipore, Billerica, MA, USA) membrane. The membrane was blocked using phosphate-buffered saline plus Tween 20 containing 5 % nonfat dry milk (w/v), and then probed with anti-β_2_-AR antibody (Santa Cruz Biotechnology, Santa Cruz, CA, USA). The bound antibody was detected using enhanced chemiluminescence (ECL; Thermo Fisher Scientific).

### Reverse transcriptase-polymerase chain reaction (RT-PCR)

Total RNA extracted from skeletal muscles using TRIzol Reagent (Invitrogen, Carlsbad, CA, USA) was reverse-transcribed using the High-Capacity cDNA Reverse Transcription Kit (Applied Biosystems, Foster City, CA, USA) with random primers. The product was subjected to PCR using Taq DNA polymerase (Takara Bio, Shiga, Japan) with the oligonucleotide primers as follows: β_2_-AR, 5′-TCT GTC TGT CTG TCT GGA TGA TG-3′ (forward), 5′-CCC ATT GTC ACA GCA GAA AGG-3′ (reverse); peroxisome proliferator-activated receptor γ coactivator 1α (PGC1α), 5′-TGA CAT AGA GTG TGC TGC TCT G-3′ (forward), 5′-TGG TTC TGA GTG CTA AGA CCG CTG-3′ (reverse); 18S, 5′-GAG AAA CGG CTA CCA CAT CC-3′ (forward), 5′-CCC AAG ATC CAA CTA CGA GC-3′ (reverse). The PCR product was electrophoresed in 1 % agarose gel (w/v) containing ethidium bromide. Fluorescence intensity was determined using the Lumino-Image Analyzer LAS-3000 System (Fuji Photo Film, Tokyo, Japan).

### Western blotting analysis for β_2_-AR signaling molecules

Total protein from skeletal muscles was extracted using Tissue Protein Extraction Reagent (Thermo Fisher Scientific), as previously described [16]. Equal amounts of protein were separated by SDS-PAGE and transferred onto a PVDF membrane (Millipore). The membrane was blocked with 5 % bovine serum albumin (w/v) in Tris-buffered saline plus Tween 20 and incubated with rabbit polyclonal antibodies against phospho-CREB (Ser133), CREB, phospho-p38 (Thr180/Tyr182), p38, phospho-Akt (Ser473), and Akt obtained from Cell Signaling Technology (Beverly, MA, USA), as well as actin antibody obtained from Santa Cruz Biotechnology; this step was followed by incubation with donkey anti-rabbit immunoglobulin G horseradish peroxidase-linked secondary antibody (GE Healthcare, Buckinghamshire, UK). Immunoreactivity was visualized using ECL (Thermo Fisher Scientific).

### Statistical analysis

Results are expressed as mean ± SEM. The significance of the difference between means was assessed by the Tukey–Kramer test after significant differences between groups were established by analysis of variance. Differences were considered significant for *P* < 0.05.

## Results

In this study, we did not observe any changes in the expression of the β_2_-AR protein in cytosol and membrane fractions, or in the expression of β_2_-AR mRNA in the soleus and TA muscles at 1–24 h after single-dose clenbuterol treatment and acute exercise (data not shown). Next, to identify β_2_-AR-induced signaling pathways that are potentially involved in skeletal muscle adaptation to single-dose clenbuterol treatment and acute exercise, we performed western blot analysis using phospho-specific antibodies against these molecules. We first investigated whether the phosphorylation of CREB in skeletal muscles is changed by single-dose clenbuterol treatment and acute exercise, but there was no change in the phosphorylation of CREB in the soleus and TA muscles at 1–24 h after single-dose clenbuterol treatment and acute exercise (data not shown).

**Fig. 1:**
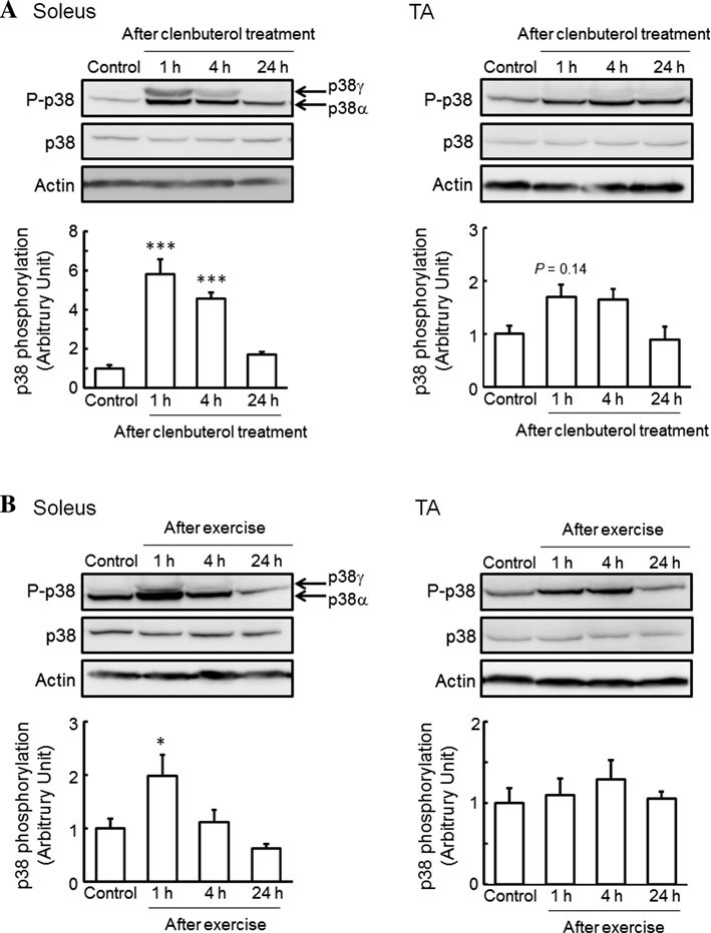
Effects of clenbuterol treatment and exercise on the phosphorylation of p38 mitogen-activated protein kinase (MAPK) in skeletal muscles. Total protein was extracted from isolated soleus and TA muscles of mice at 1, 4, and 24 h after clenbuterol treatment (1.0 mg/kg body weight) (**a**) and at 1 (just after exercise), 4, and 24 h after exercise on a treadmill for 1 h (18 m/min) (**b**), and then subjected to western blot analysis. Quantified values of western blot analysis are shown in each *bar graph*. The phosphorylation level of p38 MAPK was normalized to the abundance of p38 MAPK. Actin protein was used as a control for loading. The values shown in the *bar graphs* are relative to the optical density in control mice (set = 1). Values: mean ± SEM (*n* = 4). *P < 0.05 and ***P < 0.001 (vs. control mice)

We observed an increase in the mRNA expression of PGC1α, a regulator of adaptive thermogenesis, glucose metabolism, mitochondrial biogenesis, and muscle fiber type specialization, after single-dose clenbuterol treatment and acute exercise in the soleus and TA muscles (data not shown). It has been reported that exercise stimulates PGC1α transcription in skeletal muscles through the activation of the p38 MAPK pathway [19] and β_2_-AR [4]. These findings strongly suggest that activation of the p38 MAPK pathway mediated by β_2_-AR plays a key role in clenbuterol- and exercise-induced skeletal muscle adaptation. Therefore, we next investigated whether the phosphorylation of p38 MAPK in skeletal muscles is changed by single-dose clenbuterol treatment and acute exercise. The phosphorylation of p38 MAPK in the soleus muscle was significantly increased, to 580 and 460 %, at 1 and 4 h, respectively, after single-dose clenbuterol treatment (Fig. 1a), whereas that in the TA muscle was only relatively increased, to 170 % (*P* = 0.14) and 160 % (*P* = 0.19), at 1 and 4 h, respectively, after single-dose clenbuterol treatment (Fig. 1a). On the other hand, the phosphorylation of p38 MAPK was significantly increased, to 200 %, at 1 h after exercise in the soleus muscle, whereas that in the TA muscle remained unchanged (Fig. 1b). Furthermore, as shown in Fig. 1, a slower migrating band was observed with the phospho-specific antibody in the soleus muscle from clenbuterol-treated and exercised mice, which is indicative of phosphorylated p38γ subunits [21].

**Fig. 2:**
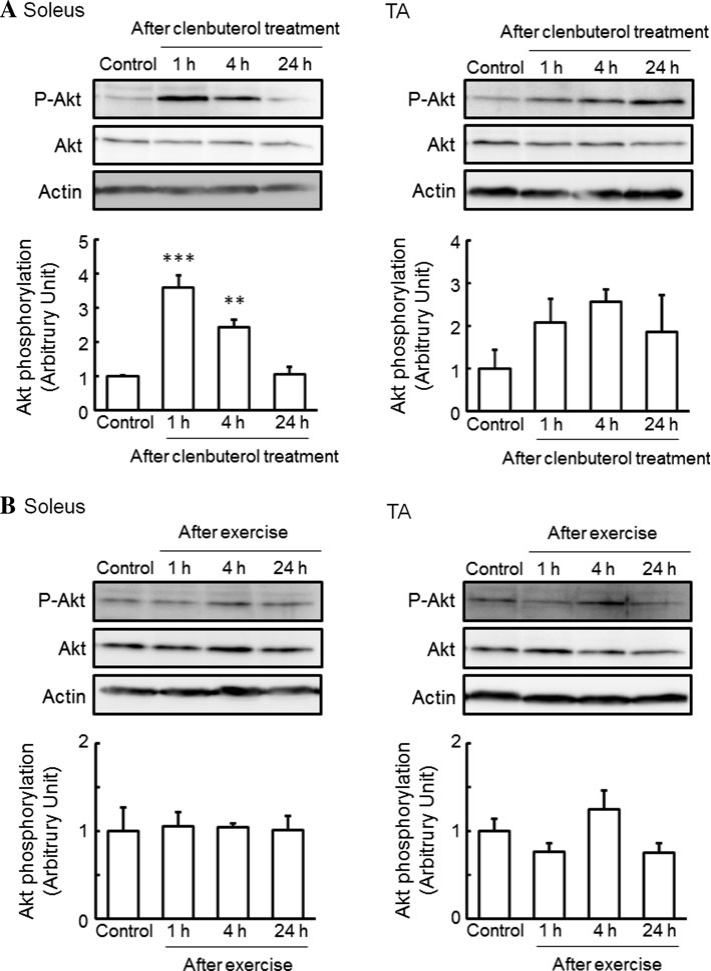
Effects of clenbuterol treatment and exercise on the phosphorylation of Akt in skeletal muscles. Total protein was extracted from isolated soleus and TA muscles of mice at 1, 4, and 24 h after clenbuterol treatment (1.0 mg/kg body weight) (**a**) and at 1 (just after exercise), 4, and 24 h after exercise on a treadmill for 1 h (18 m/min) (**b**), and then subjected to western blot analysis. Quantified values of western blot analysis are shown in each *bar graph*. The phosphorylation level of Akt was normalized to the abundance of Akt. Actin protein was used as a control for loading. The values shown in the *bar graphs* are relative to the optical density in control mice (set = 1). Values: mean ± SEM (*n* = 4). **P < 0.01 and ***P < 0.001 (vs. control mice)

Next, we investigated whether the phosphorylation of Akt in skeletal muscles is changed by single-dose clenbuterol treatment and acute exercise. The phosphorylation of Akt was significantly increased, to 360 and 240 %, at 1 and 4 h, respectively, after clenbuterol treatment in the soleus muscle, whereas that in the TA muscle remained unchanged (Fig. 2a). In contrast to clenbuterol treatment, we did not find any changes in the phosphorylation of Akt after acute exercise in the soleus and TA muscles (Fig. 2b).

## Discussion

In the present study, we observed an increase in the phosphorylation of p38 MAPK after single-dose clenbuterol treatment and acute exercise in the soleus muscle (Fig. 1), and likewise an increase in the phosphorylation of Akt after single-dose clenbuterol treatment in the soleus muscle (Fig. 2a). These results generate interest in the effects of prolonged clenbuterol treatment and exercise training on the activation of β_2_-AR signaling in skeletal muscles. Therefore, we additionally investigated whether the phosphorylation of p38 MAPK and Akt in skeletal muscles changes after 6-week clenbuterol treatment, voluntary exercise, or both. Although 6-week clenbuterol treatment specifically increased the weight of TA muscle, to 115 %, and 6-week voluntary exercise specifically increased the weight of soleus muscle, to 129 %, there was no change in the phosphorylation of p38 MAPK and Akt in the soleus or TA muscle (data not shown). These results suggest that the acute effect of clenbuterol treatment and exercise on the phosphorylation of p38 MAPK and Akt in slow-twitch muscles is transient.

The present study clearly showed that the phosphorylation of p38 MAPK in the soleus muscle was significantly increased after single-dose clenbuterol treatment and acute exercise, with an occasional increase in phosphorylated p38γ subunits (Fig. 1a). It has been demonstrated that the p38γ subunit is a key regulator in skeletal muscle metabolic adaptation [20] and required for the maintenance of slow-twitch muscle size, with no impact on fast-twitch muscles [21]. In a previous report, skeletal muscle overload was also found to increase the phosphorylation of p38 MAPK to a greater extent in slow-twitch muscles than fast-twitch muscles, with an occasional increase in phosphorylated p38γ subunits [22]. These findings suggest that the activation of p38γ MAPK after clenbuterol treatment and exercise is involved in the anabolic and/or metabolic adaptation of slow-twitch muscles.

The present study is the first report that increased phosphorylation of p38 MAPK after single-dose clenbuterol treatment is observed to a much greater extent in slow-twitch muscles than fast-twitch muscles (Fig. 1a). However, clenbuterol has been widely accepted to induce hypertrophic action against fast-twitch muscles but not slow-twitch muscles [1, 2], slow-to-fast transformation of muscle fibers, and aerobic-to-anaerobic shift of muscle metabolic enzymes [23]. Furthermore, pharmacologic blocking of p38 and JNK MAPK signaling had no effect on clenbuterol-induced fast-twitch fiber-specific gene expression [24]. From these findings, it may be concluded that clenbuterol-induced phosphorylation of p38 MAPK in slow-twitch muscles is responsible not for anabolic adaptation but for metabolic adaptation. On the contrary, it has been reported that clenbuterol increases the phosphorylation and activity of ERK1/2, members of MAPK family, to a greater extent in the TA muscle than in the soleus muscle [24]. In addition, ablation of ERK1/2 had little effect on clenbuterol-induced slow-twitch fiber-specific gene expression, whereas it inhibited clenbuterol-induced fast-twitch fiber-specific gene expression [24]. These findings suggest that ERK1/2 is necessary for the hypertrophic process in fast-twitch muscles. On the other hand, some studies have also highlighted the possibility that the activation of p38 MAPK may inhibit ERK1/2 phosphorylation [25, 26]. It is possible that the lower level of ERK1/2 activation was due to the higher level of p38 MAPK activation in the soleus muscle (Fig. 1), considering the antagonizing action of p38 MAPK on ERK1/2 activation.

Daaka et al. [6] demonstrated in the HEK293 cell line the model for β_2_-AR-mediated G-protein switching to activate MAPK; phosphorylation of MAPK by the β_2_-AR proceeds through receptor coupling to Gα_s_ and activation of PKA. Activated PKA phosphorylates the β_2_-AR leading to receptor coupling to, and activation of, Gα_i_, and then Gβγ released from the Gα_i_-coupled β_2_-AR activates MAPK in a Src- and Ras-dependent pathway. The in vitro cell culture model suggests the possibility that β_2_-agonist clenbuterol-induced receptor phosphorylation through the activation of PKA may progress in slow-twitch soleus muscle, compared to fast-twitch TA muscle, which is responsible for the increase in the phosphorylation of p38 MAPK after clenbuterol treatment to a much greater extent in the soleus muscle than the TA muscle.

Our study also demonstrated that the phosphorylation of Akt was significantly increased after clenbuterol treatment in the soleus muscle, whereas that in the TA muscle remained unchanged (Fig. 2a). Kline et al. [27] also observed an increase in the phosphorylation of Akt in rat fast-twitch muscles after single-dose and long-term (9 days) treatment with clenbuterol (3.0 mg/kg body weight). Likewise, Gonçalves et al. [28] reported that single-dose clenbuterol treatment increased the phosphorylation of Akt in rat fast-twitch muscles without changing that in rat slow-twitch muscles. In contrast to rats, single-dose treatment with clenbuterol in mice was found to increase the phosphorylation of Akt in the soleus muscle [28]. We found no change in the phosphorylation of Akt in the soleus and TA muscles after 6 weeks of clenbuterol treatment (1.0 mg/kg body weight/day) (data not shown). These findings indicate that the effect of clenbuterol on the phosphorylation of Akt differs between muscle fiber types and species, and is dependent upon the dose and duration of clenbuterol treatment.

We did not find any changes in the phosphorylation of Akt after acute exercise in the soleus and TA muscles (Fig. 2b). Some studies have shown that exercise increases the phosphorylation and activity of Akt in rat slow-twitch and fast-twitch muscles [29, 30], whereas others have observed no such effect [31, 32]. Passive stretch and electric stimulation of rat skeletal muscles was also found to increase the phosphorylation and activity of Akt in fast-twitch muscles, but not slow-twitch muscles [29, 32], whereas no such effect was observed following muscle contraction in rat skeletal muscles [31]. These findings indicate that the phosphorylation level of Akt differs between experimental models in relation to the intensity of exercise and contraction. Further experiments are needed to confirm this observation.

Specifically, in vitro cell culture experiments have revealed that the Gα_i_-linked Gβγ subunits activate the PI3K-Akt signaling pathway [7, 8, 33, 34]. Multiple skeletal muscle Akt pathways are involved not only in the signaling pathways responsible for muscle hypertrophy but also in the inhibition of signaling pathways responsible for muscle atrophy, which are activated following β_2_-AR stimulation, and these lead predominantly to hypertrophy in fast-twitch muscle [27]. Therefore, these studies have implicated that the Gβγ-PI3K-Akt signaling pathway has important roles in fast-twitch skeletal muscle hypertrophy. However, the increase in the phosphorylation of Akt following clenbuterol treatment was observed to a greater degree in slow-twitch soleus muscle than in fast-twitch TA muscle (Fig. 2a), which appears inconsistent with the finding that β_2_-AR stimulation induced hypertrophy preferentially in fast-twitch muscles [2, 14, 15]. These results indicate the possibility that activation of Akt in slow-twitch muscles may account not for anabolic adaptation but for metabolism adaptation. For instance, Akt is known to be an important signaling molecular in the insulin signaling pathways [35]. The finding that β_2_-agonist clenbuterol administration increases blood glucose and insulin concentrations [36] could be responsible for the promotion of Akt phosphorylation in skeletal muscles, where it is accepted as the best stored tissue of glycogen. Further studies are needed to clarify this hypothesis.

In conclusion, the present study shows the intracellular β_2_-AR signaling specificity in mouse skeletal muscles in response to single-dose clenbuterol treatment and acute exercise. The current findings suggest that p38 MAPK and Akt pathways play a functional role in the adaptation to clenbuterol treatment and exercise, particularly in slow-twitch muscles. In particular, the β_2_-agonists and intracellular β_2_-AR signaling have important clinical significance for enhancing muscle repair and restoring muscle function after muscle atrophy. Thus, this scientific evidence about intracellular β_2_-AR signaling in skeletal muscle could contribute to the identification of new therapeutic targets for attenuating muscle wasting and to the eradication of sports doping.
